# Influenza A Virus Defective Viral Genomes Are Inefficiently Packaged into Virions Relative to Wild-Type Genomic RNAs

**DOI:** 10.1128/mBio.02959-21

**Published:** 2021-11-23

**Authors:** Fadi G. Alnaji, William K. Reiser, Joel Rivera-Cardona, Aartjan J. W. te Velthuis, Christopher B. Brooke

**Affiliations:** a Department of Microbiology, University of Illinois at Urbana-Champaign, Champaign, Illinois, USA; b Lewis Thomas Laboratory, Department of Molecular Biology, Princeton University, Princeton, New Jersey, USA; c Carl R. Woese Institute for Genomic Biology, University of Illinois at Urbana-Champaign, Champaign, Illinois, USA; Duke University Medical Center

**Keywords:** defective interfering particles, genome packaging, influenza, sequencing

## Abstract

Deletion-containing viral genomes (DelVGs) are commonly produced during influenza A virus infection and have been implicated in influencing clinical infection outcomes. Despite their ubiquity, the specific molecular mechanisms that govern DelVG formation and their packaging into defective interfering particles (DIPs) remain poorly understood. Here, we utilized next-generation sequencing to analyze DelVGs that form *de novo* early during infection, prior to packaging. Analysis of these early DelVGs revealed that deletion formation occurs in clearly defined hot spots and is significantly associated with both direct sequence repeats and enrichment of adenosine and uridine bases. By comparing intracellular DelVGs with those packaged into extracellular virions, we discovered that DelVGs face a significant bottleneck during genome packaging relative to wild-type genomic RNAs. Interestingly, packaged DelVGs exhibited signs of enrichment for larger DelVGs suggesting that size is an important determinant of packaging efficiency. Our data provide the first unbiased, high-resolution portrait of the diversity of DelVGs that are generated by the influenza A virus replication machinery and shed light on the mechanisms that underly DelVG formation and packaging.

## INTRODUCTION

Influenza A virus (IAV) populations are highly heterogeneous and largely consist of virions that lack functional copies of one or more gene segments (1, 2). A major contributor to this heterogeneity is the common presence of defective interfering particles (DIPs) within viral populations. DIPs are virions that harbor a large deletion in one or more genome segments, resulting in an inability to express the full set of viral proteins required for productive replication. DIPs have been demonstrated in numerous contexts to interfere with wild-type (WT) virus replication (hence the name), ostensibly either by outcompeting wild-type genomes for replication and packaging and/or by triggering innate immune activation (3–7). Recent studies have correlated the abundance of DIPs within clinical samples with severity of both IAV and respiratory syncytial virus infection, suggesting a role for DIPs in modulating viral pathogenicity (8, 9). Despite being discovered over 60 years ago, the specific molecular processes that drive DIP formation, as well as the effects of DIPs on the collective behavior and pathogenicity of viral populations, remain mysterious (10, 11).

The deletion-containing genomic RNAs carried by DIPs are commonly referred to as defective viral genomes (DVGs) (3). This terminology is complicated for influenza viruses, as a variety of distinct defective viral genome species have been described, including hypermutated segments (12) and mini-viral RNAs (mvRNAs), which carry enormous deletions and do not get packaged into virions (13). In addition, it is not yet clear that the production of some deletion-containing segments is actually detrimental to viral population fitness (11). Thus, to minimize confusion, we use the term DelVG (Deletion-containing Viral Genome) here to refer to any viral gene segments that retain the classical viral packaging signals and contain deletions larger than 10 nucleotides, thus excluding small indels (14).

Next-generation sequencing (NGS) represents a powerful new tool for revealing the specific processes and molecular determinants that underly DelVG formation (15–18). The analysis of large numbers of individual DelVGs can reveal specific patterns that yield mechanistic insight into the formation process (19). IAV DelVGs are typically studied in the context of extracellular DIPs. A potential limitation of this approach for investigating the DelVG formation process is that the requirements of intracellular trafficking and packaging may specifically select for a subset of the total repertoire of DelVGs produced within the cell (20). As a result, DelVGs isolated from extracellular DIPs may not be representative of the full range of DelVG products produced within the infected cell. Such a discrepancy between the DelVGs present within an infected cell and those that get packaged into DIPs was recently described for chikungunya virus (21).

To gain a more accurate, comprehensive understanding of DelVG formation and packaging, we specifically examined the DelVGs that formed *de novo* during the first hours of IAV infection. Careful analysis of hundreds of intracellular and extracellular DelVGs revealed the enrichment of specific sequence elements at deletion breakpoints. We also observed that DelVGs represent a much larger fraction of viral RNAs within the cell compared to what gets packaged, suggesting that IAV DelVGs are packaged much less efficiently than wild-type genomic RNAs.

## RESULTS

### Intracellular DelVGs generated early during infection are primarily derived from the polymerase and HA segments.

Previous investigations of influenza DelVGs have generally focused on the RNAs that get successfully packaged into virions (DIPs). It is not actually clear how well the packaged DelVGs observed within extracellular DIPs represent the total population of DelVGs produced by the IAV replication machinery. To better understand the full array of deletions commonly generated during IAV infection, and by extension the DelVG formation process, we examined the distributions of deletion breakpoints found within intracellular viral RNAs isolated early during infection.

We infected MDCK-SIAT1 cells at a multiplicity of infection (MOI) of 10 (based on the 50% tissue culture infectious dose [TCID_50_]) with a recombinant stock of A/Puerto Rico/8/1934 (PR8) grown under low MOI conditions to minimize DIP content. We then harvested total intracellular RNA at 3 and 6 h postinfection (hpi) in order to capture the intracellular DelVGs produced early during infection. We also extracted viral RNA from supernatants (extracellular RNA) collected at 24 hpi to capture DelVGs that were successfully packaged into DIPs. These RNAs, along with genomic RNA extracted from the infecting viral stock, were used as the templates for whole-genome RT-PCR and NGS, as we have previously described (18).

**FIG 1:**
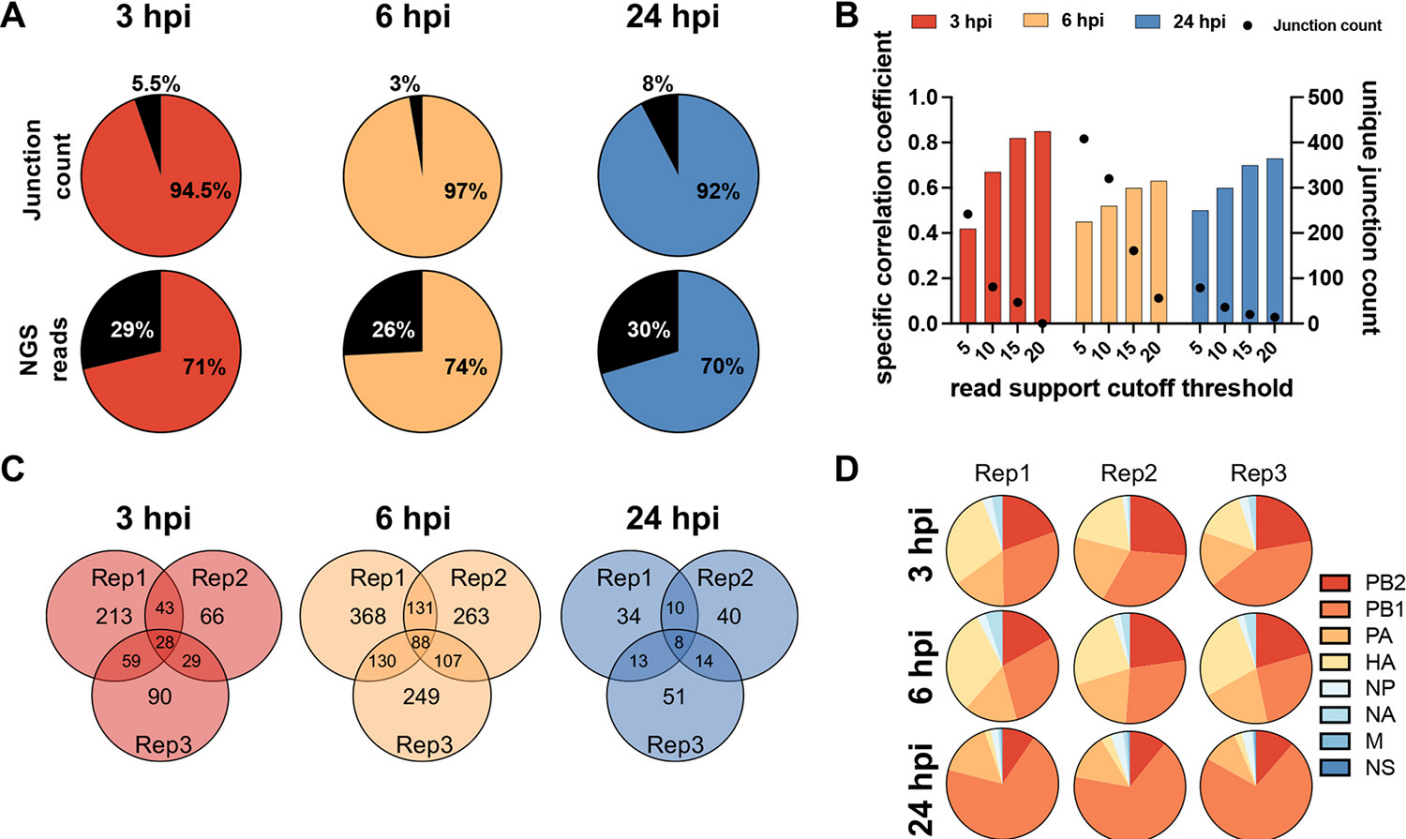
DelVG formation is partially stochastic and biased toward the polymerase and HA segments. (A) Pie charts show the proportional percentages of the total normalized junction counts and DelVG-mapping NGS read counts found in the inoculum relative to the unique junctions found in the three replicates collected at the indicated time points. The black fractions represent the junctions that were detected in the inoculum. (B) Correlation of distinct junctions in the PB2 segment between two technical replicate samples (generated from the same RNA sample) using different NGS read cutoff values. (C) Venn diagram showing the overlap in specific junctions (for the PB2 segment) between three replicate samples collected at each time point. (D) Pie charts show the proportional abundance of normalized junction counts from each genome segment across replicates. Data in this figure are from one experiment but are representative of three independent experiments.

To focus our analysis on DelVGs that formed *de novo* during the experiment, we first defined the specific DelVGs present within the inoculum and excluded them from subsequent analyses (Fig. 1A). We also only considered deletion junctions if they were represented by >5 reads within a given sample, a cutoff threshold that maximized junction detection, albeit with reduced correlation between technical replicates (Fig. 1B). We chose to prioritize junction detection sensitivity in this study due to the low copy number and read coverage of *de novo*-generated DelVGs early during infection.

Using this approach, we identified hundreds of distinct DelVGs across all segments—except M and NS—in both extracellular and intracellular samples. For each time point, we observed only partial overlap in the specific junctions shared between the three replicates, suggesting significant stochasticity in the specific locations at which deletions form (Fig. 1C). When we examined the proportional distribution of junctions across the eight genome segments, we found that these proportions were consistent across replicates, indicating significant and reproducible variation in the intrinsic potential of the individual genome segments to form DelVGs (Fig. 1D). Numerous previous studies have shown that the majority of extracellular DelVGs found within DIPs are derived from the three polymerase segments: PB2, PB1, and PA (18, 22, 23). Interestingly, we observed that hemagglutinin (HA)-derived DelVGs were roughly as numerous as polymerase-derived DelVGs at early time points within infected cells but only constituted a small fraction of extracellular DelVGs at 24 hpi, suggesting that population of DelVGs packaged into virions may not accurately reflect the relative abundances of DelVGs produced within infected cells.

### DelVG formation is not simply a function of segment length.

**FIG 2:**
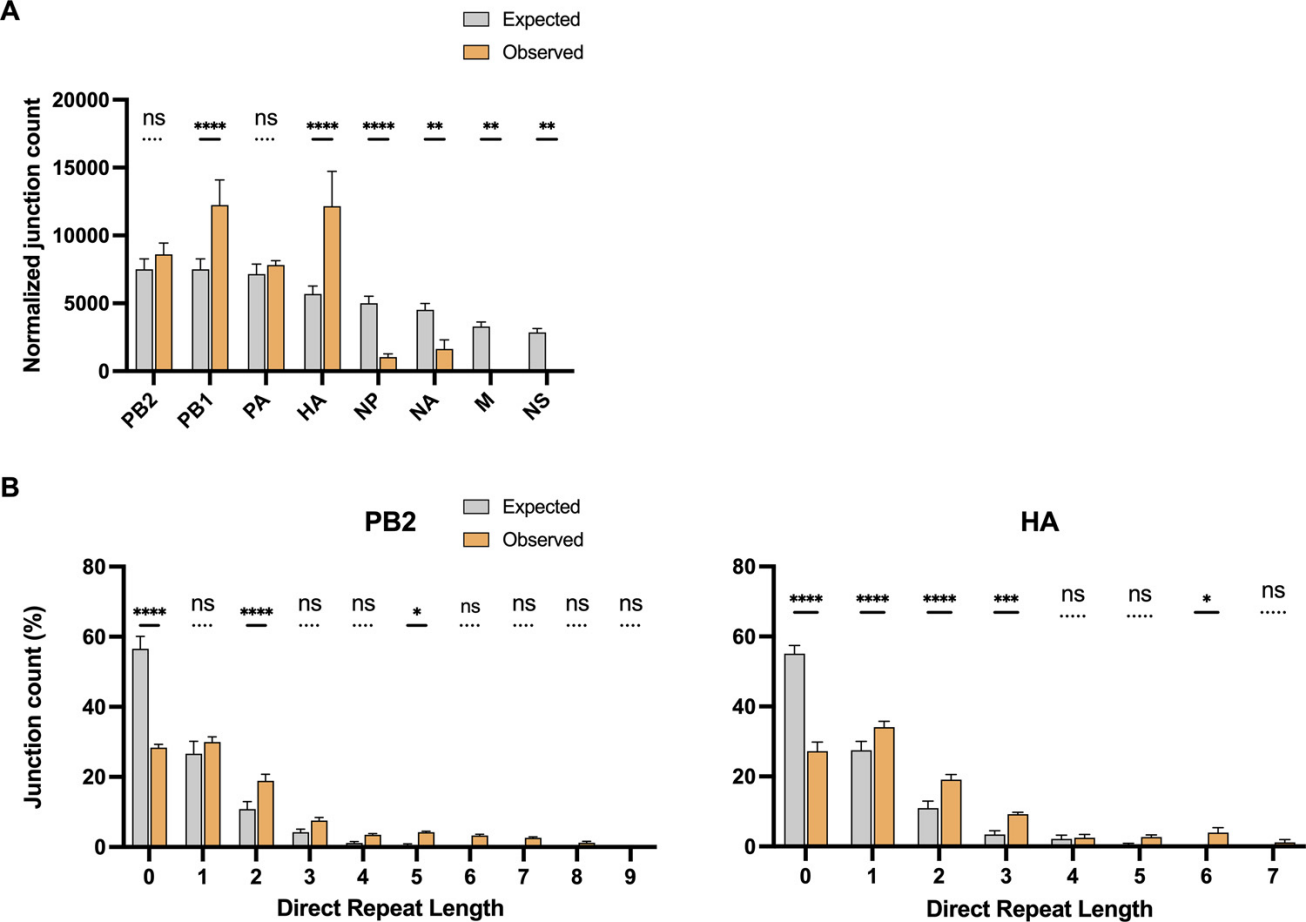
Roles of segment length and direct repeat sequences in DelVG deletion formation. (A) Observed normalized junction counts per segment from intracellular DelVGs isolated at 6 hpi were compared to junction counts predicted by a model (expected) that assumes a simple positive correlation between segment length and DelVG junction count (see Materials and Methods). (B) Numbers of intracellular DelVG junctions detected at 6 hpi with no sequence repetition flanking the deletion breakpoints (direct repeat length = 0) or with repeated sequences of various length (direct repeat length = 1 to 9) for the indicated segments (observed). Expected values plot the numbers of junctions with the indicated repeat lengths predicted from model simulations in which junction formation is random. Junction counts are plotted as a percentage of the total number of DelVGs detected for a given segment. The data are presented as means (*n* = 3 cell culture wells) ± the standard deviations. *, *P* < 0.05; **, *P* < 0.01; ***, *P* < 0.001; ****, *P* < 0.0001; ns, not significant (two-way ANOVA).

It is not clear why some segments are more prone to DelVG formation than others, though it has been postulated that this is simply a function of the segment length (24). To directly test this hypothesis, we compared the normalized junction counts of intracellular DelVGs detected at 3 and 6 hpi (which should represent newly formed DelVGs) with a perfect model that assumes a positive correlation between junction count and the proportional length of each segment (Fig. 2A; see also Fig. S1A). Although the PB2 and PA segments matched up well with the model predictions, the other segments deviated significantly. We observed similar results at 24 hpi (see Fig. S1B). These data demonstrate that rates of DelVG formation are not proportional to segment length.

10.1128/mBio.02959-21.1 FIG S1 Segment length and direct repeat sequences in DelVGs. (A) Observed normalized intracellular junction counts per segment at 3 hpi were compared to junction counts predicted by a model (expected) that assumes a simple positive correlation between segment length and DelVG junction count (see Materials and Methods). (B) Same as panel A but at 24 hpi. (C) Numbers of intracellular DelVG junctions detected at 3 hpi with no sequence repetition flanking the deletion breakpoints (direct repeat length = 0) or with repeated sequences of various length (direct repeat length = 1 to 9) for the indicated segments (observed). Expected values plot the numbers of junctions with the indicated repeat lengths predicted from model simulations in which junction formation is random. (D) Same as panel C but at 24 hpi. Junction counts are plotted as a percentage of the total number of DelVGs detected for a given segment. The data are presented as means (*n* = 3 cell culture wells) ± the standard deviations. * *P* < 0.05; ** *P* < 0.01; ***, *P* < 0.001; ****, *P* < 0.0001; ns, not significant (two-way ANOVA). Download FIG S1, TIF file, 0.9 MB. Copyright © 2021 Alnaji et al. 2021 Alnaji et al. https://creativecommons.org/licenses/by/4.0/ This content is distributed under the terms of the Creative Commons Attribution 4.0 International license.

### Direct sequence repeats and A/U bases are enriched at DelVG deletion junctions.

By examining newly produced DelVGs isolated early during infection, we generated an unbiased view of DelVG deletion formation by the IAV replicase. Previous reports have described an enrichment of repeated sequences flanking DelVG deletions (termed “direct repeats”) and have hypothesized these direct repeats may promote DelVG formation by facilitating RNA-dependent RNA polymerase (RdRp) reengagement during the replication process (16, 25). In support of these previous studies, in intracellular DelVGs collected at early time points we observed that deletions lacking direct repeat sequences were significantly less abundant and that a subset of direct repeat sequence lengths were more abundant than would be predicted if deletions were completely random (Fig. 2B; see also Fig. S1C). Similar results were seen in extracellular DelVGs at 24 hpi (see Fig. S1D).

**FIG 3:**
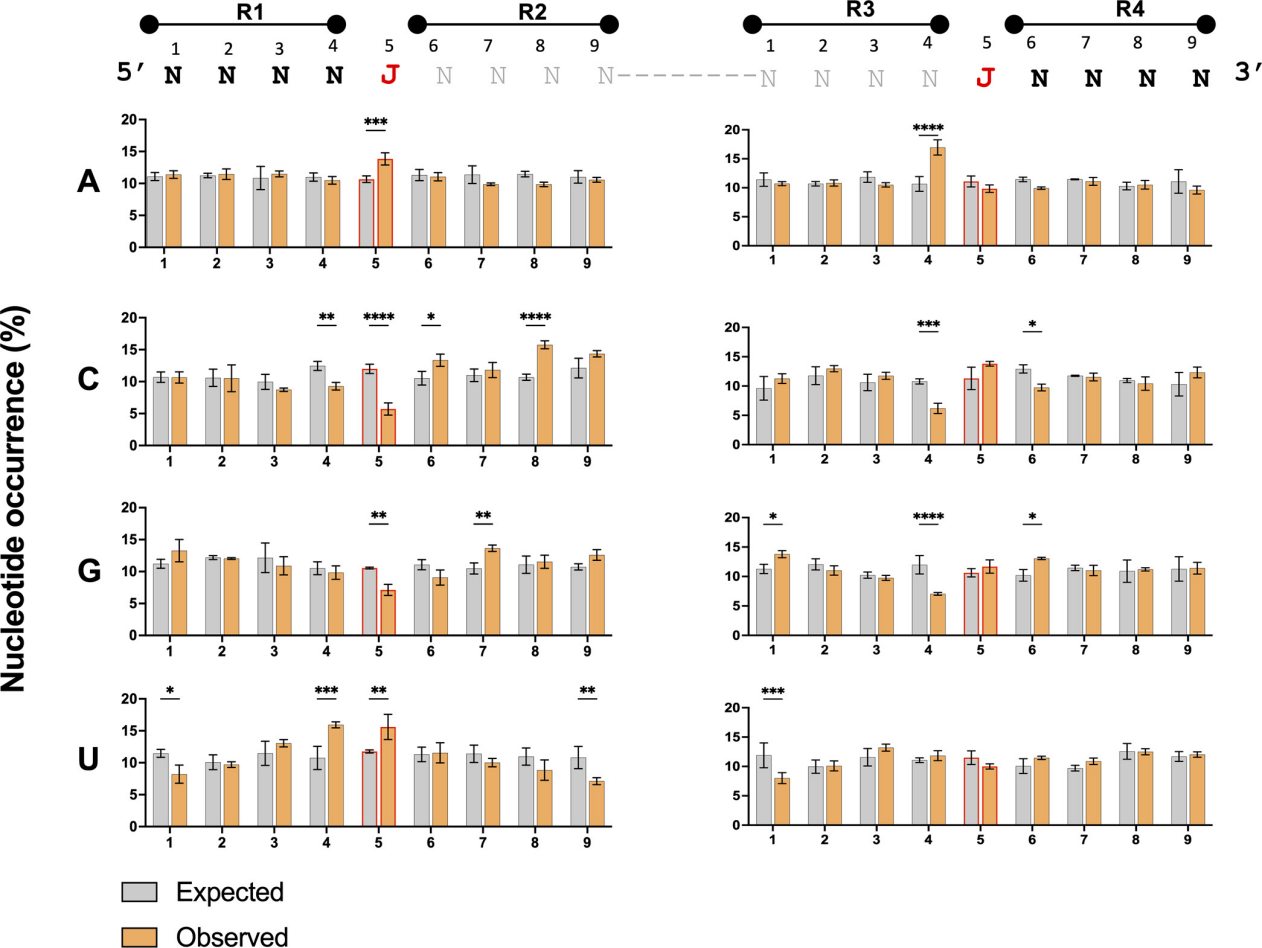
Enrichment of specific nucleotides at positions flanking DelVG deletions. The 4 nucleotides flanking DelVG junctions were numbered and divided into four regions (R1 to R4), and the percentage occurrence of each nucleotide was calculated at each site within each region. The junction nucleotides immediately flanking the deletion are indicated by the red “J” ’s at position 5 on the left and right. The gray nucleotides flank the junctions in the progenitor sequence but are lost through deletion in the actual DelVG sequence. The percentage of nucleotide occurrence (observed in intracellular PB2 DelVGs collected at 6 hpi) at each site was plotted against a random control (expected). The data are presented as means (*n* = 3 cell culture wells) ± the standard deviations. *, *P* < 0.05; **, *P* < 0.01 ***, *P* < 0.001; ****, *P* < 0.0001 (two-way ANOVA); ns, not significant.

We also sought to determine whether specific sequence motifs were enriched at DelVG deletion junctions. Using the intracellular samples from 6 hpi, we extracted the sequences flanking each deletion breakpoint in the predeletion, wild-type sequence and calculated the proportion of each nucleotide at each position (Fig. 3). To determine whether the observed nucleotide frequencies deviated from what would be expected if deletion formation was sequence independent, we performed the same analysis on three replicate sets of deletions randomly generated *in silico*. Comparison of the observed nucleotide frequencies with the null model predictions revealed clear enrichment of adenosines or uridines at the 5′ deletion breakpoint position (labeled “J” in Fig. 3). We also detected enrichment of either adenosines or uridines at the position immediately upstream of both the 5′ and 3′ breakpoints (position 4 in both R1 and R3). Finally, we observed enrichment of either cytidines or guanosines within the R2 region downstream of the 5′ breakpoint. Altogether, these results suggest that DelVG formation may be facilitated in part by the presence of direct sequence repeats and nucleotide composition surrounding the junction sites.

### Intracellular DelVGs are inefficiently packaged.

**FIG 4:**
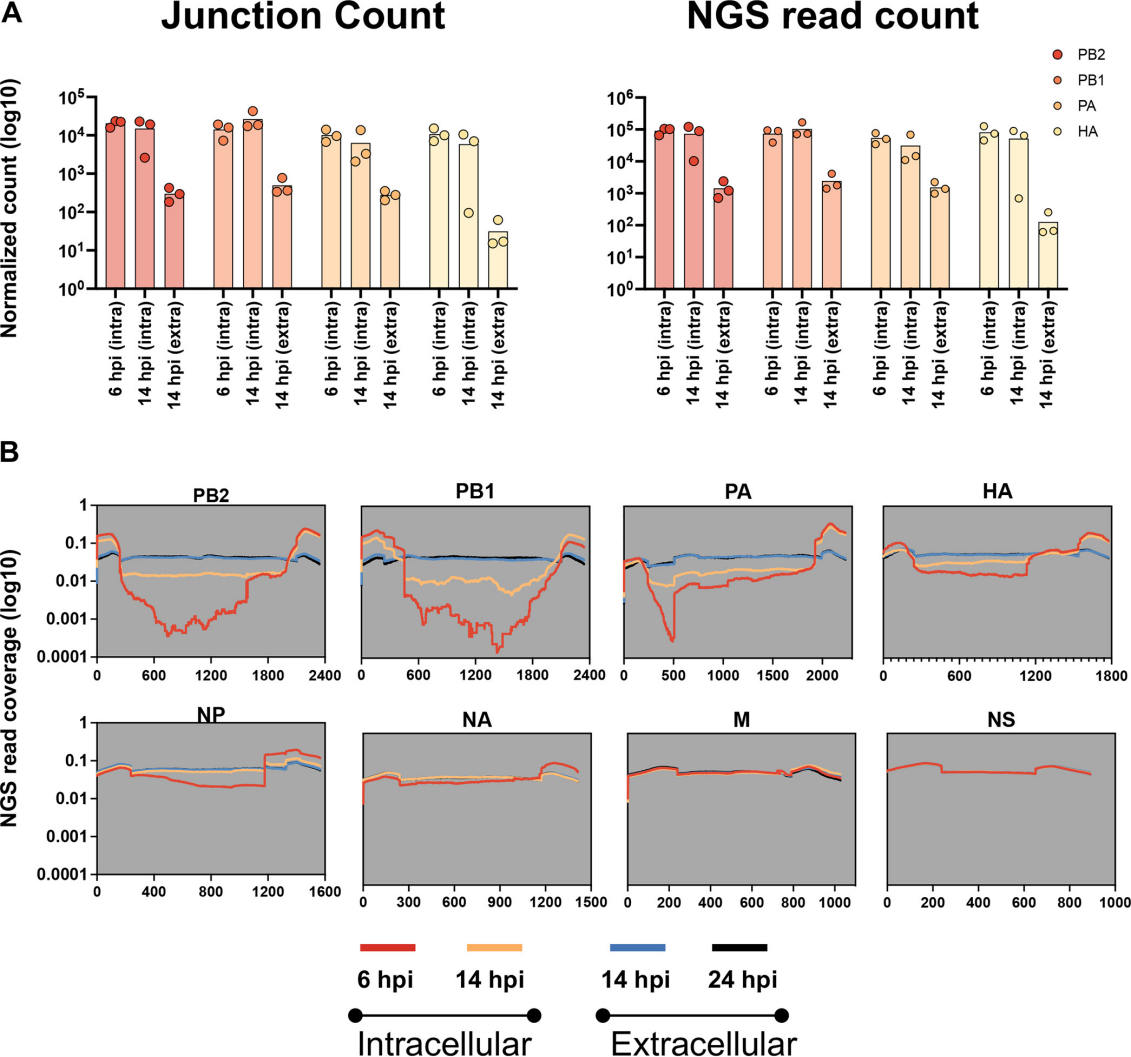
Lower representation of DelVGs extracellularly than intracellularly. (A) Numbers of DelVG junction counts (left panel) and NGS reads (right panel) were counted for three replicates in each segment at each indicated time point, normalized to 106 mapped reads. Each dot represents a replicate. (intra = intracellular, extra = extracellular). (B) The NGS read coverage per nucleotide was plotted for each segment at each time point (the per nucleotide coverage was normalized to the total number of DelVG reads of each segment at each replicate before plotting the average of the three replicates).

Our finding that HA-derived DelVGs make up a large fraction of intracellular but not extracellular DelVGs suggested that some DelVGs might be packaged into virions more efficiently than others. This was also verified by comparing the normalized DelVG junction and NGS counts between the three time points (see Fig. S2). To further investigate this, we repeated the experiment under the same conditions and compared both the normalized numbers of unique junctions and NGS read support for DelVGs from each segment between intracellular (6 and 14 hpi) and matched extracellular (14 hpi) populations (Fig. 4A; see also Fig. S3).

10.1128/mBio.02959-21.2 FIG S2 Extracellular viral RNA exhibits lower DelVGs representation compared to intracellular viral RNA (first experiment). Numbers of DelVG junction counts (upper panel) and DelVG-mapping NGS reads (lower panel) of samples were counted for three replicates in each segment at each indicated time point, including the inoculum, normalized to 10^6^ mapped reads. Each dot represents a replicate. Download FIG S2, TIF file, 0.8 MB. Copyright © 2021 Alnaji et al. 2021 Alnaji et al. https://creativecommons.org/licenses/by/4.0/ This content is distributed under the terms of the Creative Commons Attribution 4.0 International license.

10.1128/mBio.02959-21.3 FIG S3 Extracellular viral RNA exhibits lower DelVGs representation compared to intracellular viral RNA (repeat experiment). Data from a repeat of the experiment depicted in Fig. S2 are shown. Download FIG S3, TIF file, 0.6 MB. Copyright © 2021 Alnaji et al. 2021 Alnaji et al. https://creativecommons.org/licenses/by/4.0/ This content is distributed under the terms of the Creative Commons Attribution 4.0 International license.

While normalized DelVG junction count numbers within intracellular RNA samples were similar for individual segments between 6 and 14 hpi, we observed an ∼100-fold decrease in normalized junction counts in extracellular samples taken at 14 hpi (Fig. 4A, left panel). The same trend was also observed when we compared DelVG NGS read support across time points (Fig. 4A, right panel). These data indicate that DelVGs constitute a much smaller fraction of total viral RNAs in extracellular virions compared their proportion within the infected cell.

To confirm this observation, we examined the per-nucleotide read coverage for each segment. Since DelVGs are missing large internal regions of the genomic RNA sequence but still retain the segment termini, they produce a characteristic “devil horns” read coverage pattern, where read coverage is substantially lower in the middle of gene segment compared to the termini (26). This pattern was obvious for segments 1 to 4 in intracellular samples at 6 hpi and for segments 1 to 3 at 14 hpi (Fig. 4B). In contrast, read coverage was consistent across the length of all eight segments in extracellular samples at 14 and 24 hpi, providing further evidence that DelVGs make up a much larger fraction of total viral RNA inside the infected cell compared to that which gets packaged and released.

To directly quantify the relative packaging efficiencies of DelVGs and WT vRNAs, we performed an *in vitro* competition assay between WT PR8 and recombinant stocks of two different DIPs that harbor distinct internal deletions in their PB2 segment: DI244 and DI291. DI244 is a well-characterized DIP/DelVG that harbors a 1,946-nucleotide deletion (27, 28), while DI291 has not been previously described and retains the 5′ 333 nucleotides and the 3′ 291 nucleotides of the PB2 segment. We coinfected MDCK-SIAT1 cells in triplicate with a 1:1 ratio (based on PB2 gene equivalents) of WT PR8 and DI244 or DI291 at a combined multiplicity of infection (MOI) of 20 PB2 gene equivalents/cell under single cycle conditions where secondary spread was not permitted. Finally, we quantified the copy number of each virus using qPCR primer/probe sets specific for either the WT, DI244, or DI291 versions of the PB2 gene segment. We confirmed that the absolute copy numbers of both WT and DI244 or DI291 PB2 gene equivalents present in the inoculum were equivalent (see Fig. S4A).

**FIG 5:**
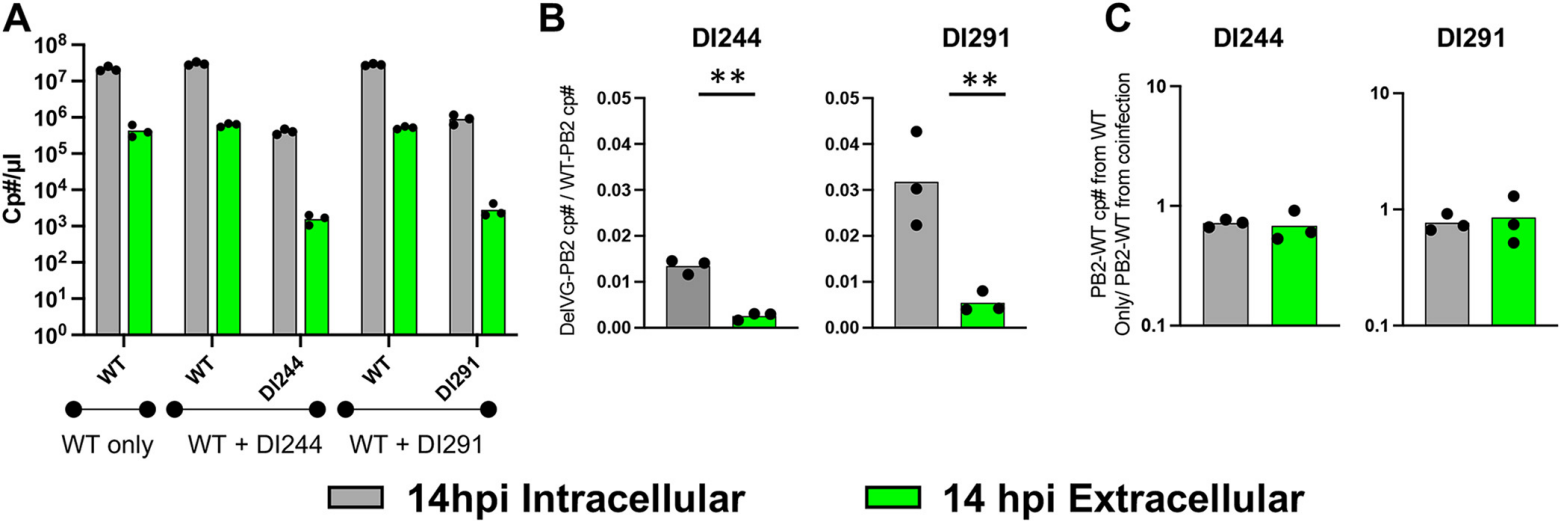
DelVGs are inefficiently packaged relative to WT vRNAs. (A) *In vitro* competition assay between the WT and one of two recombinant DIPs: DI244 or DI291. MDCK-SIAT1 cells were coinfected with WT PR8 only, WT+DI244, or WT+DI291 at a 1:1 ratio for a final MOI of 20 PB2 gene equivalents/cell under single cycle conditions. Absolute copy numbers of WT, DI244, and DI291 PB2 segments were quantified by RT-qPCR in both intracellular and extracellular RNA samples isolated from the same cell culture wells at 14 hpi. (B) The ratio of DelVG-derived to WT PB2 genome equivalents was determined in both intracellular and extracellular RNA samples collected at 14 hpi from WT-DIP coinfections. (C) Fold change in extracellular WT PB2 genome equivalents produced under WT-only infection conditions versus WT-DIP coinfection conditions. The data are presented as means (*n* = 3 cell culture wells). **, *P* < 0.01 (*t* test; cp#/μl = cDNA copy number per μl).

10.1128/mBio.02959-21.4 FIG S4 Control samples for the *in vitro* competition assay experiment. (A) qPCR quantification for WT, DI244, and DI299 in the inoculum of the experiment shown in Fig. 5. Each dot represents a qPCR technical replicate. (B) MDCK-SIAT1 cells were infected with either DI244 only or DI299 only; viral RNA was harvested intra- and extracellularly at 14 hpi, and TaqMan qPCR was finally used to quantify the PB2 segment of both DelVGs using specific primers/probe. The data are presented as means (*n* = 3 cell culture wells). Download FIG S4, TIF file, 0.2 MB. Copyright © 2021 Alnaji et al. 2021 Alnaji et al. https://creativecommons.org/licenses/by/4.0/ This content is distributed under the terms of the Creative Commons Attribution 4.0 International license.

As a negative control, we infected MDCK-SIAT1 cells with only DI244 or DI291 (no WT virus) and failed to observe any signs of active replication, as expected (see Fig. S4B). As a positive control, we infected MDCK-SIAT1 cells with WT only (no DIPs) at an MOI of 10. For the WT-DIP coinfections, we quantified the amounts of WT-, DI244-, and DI291-derived PB2 genome equivalents in both intracellular and extracellular samples collected from the same cells at 14 hpi (Fig. 5A). Within infected cells at 14 hpi, WT PB2 was ∼80- and ∼30-fold more abundant than DI244- and DI291-derived PB2, respectively (Fig. 5B), suggesting that the WT PB2 segment enjoys a significant replicative advantage over both DelVGs during the first 14 h of infection. This difference was significantly (*t* test, *P* < 0.01) more pronounced in extracellular RNA collected at 14 hpi, where WT PB2 outnumbered DI244- and DI291-derived PB2 ∼400- and ∼200-fold, respectively. The significantly decreased proportional abundance of PB2 DelVGs relative to WT PB2 in extracellular RNA compared to intracellular RNA observed in these coinfection experiments further supports the conclusion that DelVGs are packaged less efficiently than WT vRNAs. Finally, we observed no differences in either intracellular or extracellular levels of WT RNA between the WT-only infection and WT/DIP coinfections (Fig. 5C), suggesting that WT RNA replication and packaging are not influenced by the presence of DelVGs under these experimental conditions.

Collectively, these data demonstrate that DelVGs make up a much smaller fraction of packaged viral RNAs compared their proportion of viral genomic RNAs within the infected cell, consistent with a packaging defect relative to WT vRNAs.

### DelVGs packaging is biased toward longer DelVGs.

The discrepancies that we observed in both proportional abundance and distribution among the segments between intracellular and extracellular DelVGs suggested the existence of a significant bottleneck limiting packaging of DelVGs relative to wild-type vRNAs. We hypothesized that this might be due to the potential loss of sequences required for efficient packaging during the formation of some DelVGs. If true, we expected that specific regions of gene segments required for maximal packaging efficiency would be retained within packaged extracellular DelVGs but largely missing from intracellular DelVGs.

**FIG 6:**
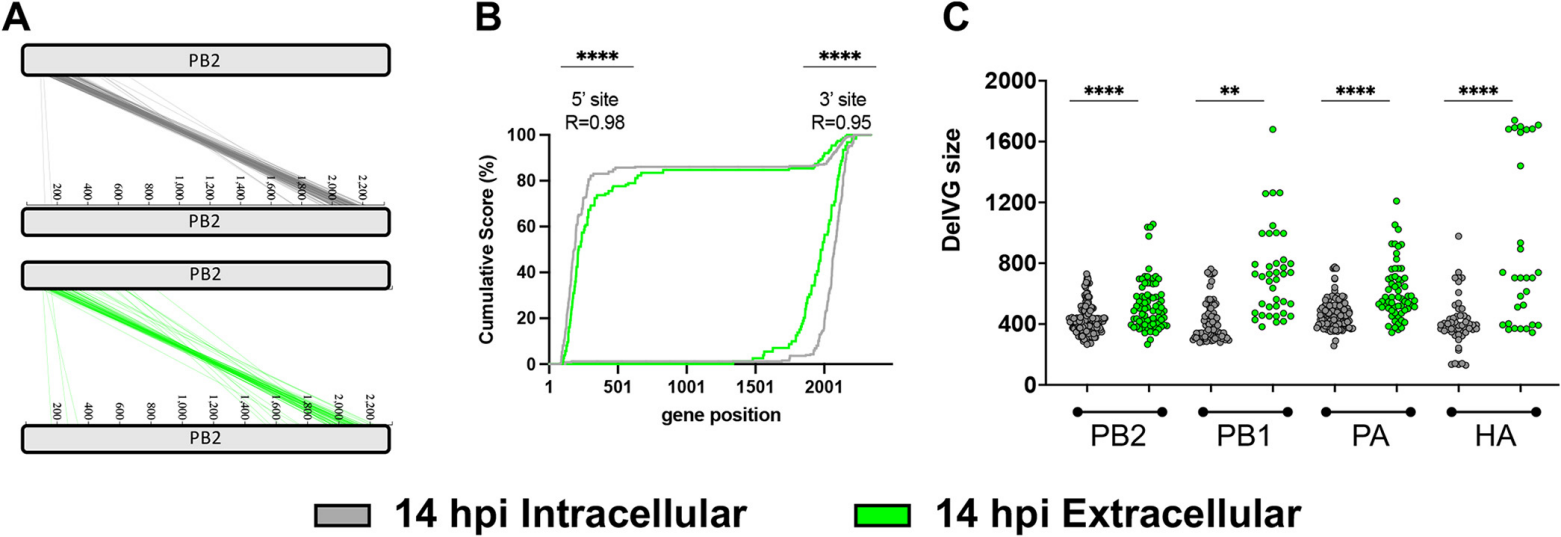
Longer DelVGs are enriched in the extracellular viral RNA (A) Deletion junction sites in intracellular and extracellular PB2-derived DelVGs collected at 14 hpi were mapped to their genome positions with each diagonal line representing a distinct DelVG junction. (B) Plots show the cumulative occurrence of DelVG deletions as a function of gene segment position of segment PB2. Cumulative score was calculated by starting at zero at the end of the segment and then adding a score of 1 at each nucleotide where a unique junction breakpoint occurred. Scores were normalized by calculating the percentage of final value reached at each position. Pearson correlation coefficient *R* and *P* values are shown for both 5′ and 3′ junction sites. (C) DelVG size distributions from the first four genome segments. The data are presented as means (*n* = 3 cell culture wells) ± the standard deviations. **, *P* < 0.01; ****, *P* < 0.0001 (*t* test).

To test this, we first examined the positions of intracellular and extracellular DelVG deletion junctions from at 14 hpi (Fig. 6A; see also Fig. S5A). As we and others have previously reported for packaged extracellular DelVGs, deletion junctions from both populations clustered within clear hot spots near the segment termini (26, 29, 30). To more rigorously evaluate whether the locations of deletion junctions differed between intracellular and extracellular DelVGs, we used a cumulative score method that allowed us to examine the proportional distributions of deletion junctions as a function of nucleotide position at both the 5′ and 3′ ends of gene segments (Fig. 6B; see also Fig. S5B). We observed a significant correlation in the distributions of 5′ and 3′ junction locations between intracellular and extracellular DelVGs (*R* ≈ 0.96, *P* < 0.0001), suggesting no significant differences between the populations. However, the deletion junctions appeared to skew more toward the interior of the segment for the extracellular DelVGs, which led us to ask whether the size of intracellular and extracellular DelVGs varied at 14 hpi (Fig. 6C). Across the first four segments, we observed a significant bias toward longer DelVGs in the extracellular compartment compared to intracellular RNAs (*t* test, *P* < 0.01 to 0.001), indicating that DelVG size correlates with packaging efficiency.

10.1128/mBio.02959-21.5 FIG S5 Distribution of DelVG junctions show clear clustering toward the termini. (A) The intracellular and extracellular PB1- and PA-derived DelVG deletion junction sites at 14 hpi were mapped to their genome positions with each line representing a distinct DelVG and the colors indicating whether they are from intracellular or extracellular samples. (B) Plots show the cumulative occurrence of DelVG deletions as a function of gene segment position of segment PB2 at both 3′ and 5′ sites. The cumulative score was calculated by starting at zero at the end of the segment and then adding a score of 1 at each nucleotide where a unique junction breakpoint occurred. Scores were normalized by calculating the percentage of final value reached at each position, and finally the average score of the three replicates was plotted per position. Person correlation coefficient *R* and *P* values are placed for both 5′ and 3′ sites (****, *P* < 0.0001). Download FIG S5, TIF file, 0.4 MB. Copyright © 2021 Alnaji et al. 2021 Alnaji et al. https://creativecommons.org/licenses/by/4.0/ This content is distributed under the terms of the Creative Commons Attribution 4.0 International license.

## DISCUSSION

Despite recent improvements in our fundamental understanding of the structure and function of the IAV polymerase complex, we still do not know how or why DelVGs and DIPs form during IAV replication (11, 31–34). Here, we used a robust combined NGS/analysis pipeline to analyze the first wave of DelVGs that form within cells during infection, thus providing the first unbiased view of *de novo* DelVG production by the IAV replicase. We compared these intracellular DelVGs to the population of DelVGs that get packaged into virions, revealing a significant bottleneck in DelVG packaging relative to wild-type vRNAs. Our data contradict the dogma that DelVGs outcompete wild-type vRNAs for packaging and suggest that the commonly observed ability of DIPs to outcompete WT virus over multiple generations must arise from other mechanisms.

In agreement with previous studies, we found that the majority of extracellular DelVG junctions were derived from the three polymerase segments (25). Within intracellular DelVG populations however, the abundance of HA-derived DelVGs was comparable to that of the polymerase-derived DelVGs, suggesting that DelVGs from segments 1 to 4 are generated at similar rates but that the HA segment packaging efficiency is much more sensitive to deletions than the polymerase segments. It has been suggested that this bias in DelVG formation across the segments is a function of segment length, potentially because DelVGs derived from longer segments have a greater length differential compared to their wild-type parents, resulting in a more pronounced replication advantage (3, 35). Our findings that the enrichment of DelVGs from segments 1 to 4 is already apparent by 3 hpi suggests that this bias is emerging from the formation process rather than replication. More work is needed to identify the specific determinant(s) that influence the uneven distribution of DelVG formation across genome segments.

DelVG formation is thought to occur when the viral replicase pauses synthesis of the daughter vRNA or cRNA but continues processing along the template and reinitiates synthesis at a downstream site on the same template (3, 36). This process is not completely random, since the vast majority of deletions start and stop within hot spots near the segment termini, and individual segments vary greatly in DelVG formation (25, 26, 37). We observed the same distribution in intracellular DelVGs, indicating that these hot spots reflect that what is produced by the viral RdRp and are not biased by selection in the packaging process. Altogether, our data suggest that specific regions of the viral genome are uniquely prone to DelVG formation, for reasons that are still not understood.

It has been suggested that the polymerase translocation is promoted by the presence of a direct sequence repeat and a specific nucleotide composition at the junction site (6, 16, 25). We demonstrate significant enrichment of direct sequence repeats and A/U nucleotides at DelVG deletion junctions, along with C/G nucleotides downstream of the 5′ deletion breakpoint. Interestingly some of these nucleotides are located within the regions that are not retained in DelVG final product (R2 and R3 in Fig. 3), suggesting possible roles for the sequence composition both upstream and downstream of junction sites at both ends of the viral genome. These specific template sequence features likely enhance the probability of RdRp translocation occurring; however, these features are not absolutely required, as large numbers of DelVG junctions that lack flanking direct repeats or A/U bases can easily be observed. There also appears to be a significant degree of stochasticity in the specific nucleotides at which deletions form based on the limited degree of overlap in breakpoint locations between replicates.

DelVGs/DIPs are known for their ability to inhibit the replication of WT virus, and it is widely believed that this effect is partially driven by DelVGs outcompeting WT vRNAs for packaging (38). Our data strongly suggest that the opposite is true: that DelVGs are inefficiently packaged into virions compared with WT vRNAs. This finding complicates our understanding of how DIPs outcompete WT virus at the population level, suggesting that other advantages must help DelVGs/DIPs offset their packaging deficiencies.

IAV genome packaging is governed by multiple, discontinuous regions that act in *cis* and in *trans* to facilitate the efficient and selective incorporation of a single copy of each genome segment into the vast majority of virions (30). Each segment contains packaging and bundling sequences that span both coding and noncoding regions of the segment termini (39–43). Beyond the well-described packaging signals in the segment termini, additional packaging determinants exist within the interiors of some segments (44–47). Consistent with this, we observed a significant bias toward shorter deletions in successfully packaged DelVGs versus intracellular DelVGs, indicating that the retention of longer terminal sequences is associated with more efficient packaging. These data suggest that the relative inefficiency of DelVG packaging stems from the loss of sequence elements required for optimal packaging efficiency.

Our analysis of the initial wave of DelVGs produced *de novo* during IAV infection provides critical insights into the formation and packaging of both DelVGs and WT RNAs. This approach generated a detailed portrait of the full range of DelVG produced by the IAV replicase, allowing us to show that DelVGs are inefficiently packaged into particles relative to WT, contrary to dogma. In addition, we showed that DelVG formation is not influenced by the segment length but is partially influenced by sequence context. Several fundamental questions remain, however, including the specific mechanism that triggers DelVG formation, the factors that cause DelVGs to form at defined hot spots and in higher frequency within some segments versus others, and whether and how DelVG formation can be modulated by the host cell environment or viral genotype.

## MATERIALS AND METHODS

### Virus and cells.

MDCK-SIAT1 and HEK293T cells were grown in minimal essential medium (MEM) plus GlutaMAX (Gibco), supplemented with 8.3% fetal bovine serum (FBS; Seradigm), at 37°C and 5% CO_2_. Recombinant A/Puerto Rico/8/1934 (PR8) was generated from HEK293T cells through standard influenza virus 8-plasmid reverse-genetics transfection. Undiluted transfection supernatants were directly inoculated onto MDCK-SIAT1 cells, and supernatants were harvested at the first signs of cytopathic effect to generate seed stocks. Working stocks of virus were generated by infecting MDCK-SIAT1 cells with seed stock at an MOI of 0.0001 TCID_50_/cell and harvesting at 48 hpi. The supernatant was clarified at 3,000 rpm for 5 min, and 500-μl aliquots were stored at −70°C.

### Generation of DelVG through high-MOI infection.

Confluent MDCK-SIAT1 were infected in triplicate with PR8 at an MOI of 10 TCID_50_/cell. To harvest intracellular viral RNA at 3, 6, and 14 hpi, cells were washed twice with phosphate-buffered saline, and RNA was extracted using a Qiagen RNeasy kit according to the manufacturer’s instructions. To extract extracellular RNA from packaged virions, the supernatant was collected from infected cells at 14 and 24 hpi, clarified, and incubated for 30 min with RNase A (0.25 μg). Next, 140 μl of supernatant was used for RNA extraction using the Qiagen QIAamp viral RNA minikit according to the manufacturer’s instructions. All RNA was stored at −70°C.

### Viral cDNA amplification and sequencing.

Universal RT-PCR was performed on all the samples before sequencing on Illumina MiSeq or NovaSeq using a previously described method (18, 48).

### Generation of recombinant DI244 and DI291.

We synthesized (Integrated DNA Technologies, Inc.) and cloned the full-length DI244 (NCBI L41510.1) or DI291 sequence into the pDZ vector and transfected it along with seven plasmids encoding WT versions of segments 2 to 8 plasmids into PB2-expressing HEK293 cells (HEK293-PB2), using a standard eight-plasmid reverse-genetics approach, as previously described (18). Transfection supernatant was used to infect PB2-expressing MDCK cells (MDCK-PB2) for 48 h to generate a seed stock. HEK293-PB2 and MDCK-PB2 cells were kindly provided by Stefan Pöhlmann and were as described previously (5). Both DelVG sequences were confirmed by deep sequencing.

### RT-qPCR quantification of DI244, DI291, and WT PB2 gene segments.

We designed and optimized specific primer/probe sets (see Table S1) specific for either DI244, DI291, or WT PB2 using the IDT PrimerQuest webtool and validated efficiency and specificity using serial dilutions of plasmids encoding either WT PB2, DI291, or DI244. Viral RNA was extracted from cells or virions as described above and used to synthesize cDNA using the universal primer and a Verso cDNA synthesis kit (Thermo Fisher). First, 3 μl of RNA was mixed with 8 μl of H_2_O, 4 μl of 5× cDNA synthesis buffer, 2 μl of dNTP mix (5 mM each), 1 μl of universal primer (10 μM), 1 μl of RT enhancer, and 1 μl of Verso enzyme mix, before incubation for 50 min at 45°C. After this, 1 μl of the of cDNA product was mixed with 7 μl of H_2_O, 1 μl of forward primers (18 μM), 1 μl of reverse primer (18 μM), 1 μl of specific probe (5 μM), and 10 μl of TaqMan Fast Advanced Master Mix (Thermo Fisher). The qPCR conditions used were as follows: 50°C (2 min) and 95°C (2 min), followed by 40 cycles of 95°C (1 s) and 61°C (20 s) using a qPCR QuantStudio 3 thermocycler.

10.1128/mBio.02959-21.6 TABLE S1 qPCR primers and probes used for the competition assay. Download Table S1, PDF file, 0.05 MB. Copyright © 2021 Alnaji et al. 2021 Alnaji et al. https://creativecommons.org/licenses/by/4.0/ This content is distributed under the terms of the Creative Commons Attribution 4.0 International license.

### *In vitro* competition assay.

MDCK-SIAT1 cells were coinfected in duplicate at an MOI of 10 PB2 gene equivalents/cell with a 1:1 ratio of WT PR8 and DI244 or DI291 (ratio based on PB2 gene equivalents). Fractions of the inoculum mixture before (0 hpi) was set aside for RT-qPCR. After adsorption for 1 h at 4°C, inoculum was removed, cells were washed, and MEM+FBS was added to the cells. At 3 hpi, neutralizing anti-HA monoclonal antibody H36-26 was added to each well to a final concentration of 25 μg/ml to block secondary spread. Intracellular and extracellular RNA was extracted as described above.

### Sequencing analysis of deletion junctions.

Raw sequencing reads were fed into our DI-detection pipeline for junction detection and characterization, as previously described (18). To account for sequencing read coverage variations between libraries, we normalized the junction count and their NGS reads to 10^6^ mapped reads per library per segment. For the junction count, the number of junctions per segment was multiplied by 10^6^ and divided by the number of NGS reads aligned to the WT gene of the given segment in a given library. The same was done to normalize for the NGS reads, where the junctions’ read number per segment was multiplied by 10^6^ and divided by the number of NGS reads aligned to the WT genome of the given segment plus the number of reads mapped to the junctions.

The direct repeat sequence lengths were extracted from the output file “Virus_Recombination_Results.txt” generated by ViReMa algorithm. For the random control, the junction sites were randomized using Excel function “=RANDBETWEEN()” based on the actual sequence range and number detected in the 6-hpi population. Next, a custom Perl code was used to extract their sequences from the corresponding PR8 gene segment (PB2 and HA). Finally, the direct repeat lengths were extracted and compared to the real samples.

To analyze the nucleotide composition at the junction site, we analyzed the sequences flanking the junction sites for enrichment of specific nucleotides. There are four possible sequence regions that possibly involved in promoting the polymerase translocation, two regions flanking the junction from each site, we numbered them regions 1 to 4 (R1 to R4) (Fig. 3). From these regions only, R1 and R4 are retained within the DelVG final product, while R2 and R3 are not, and their potential importance stems from their physical proximity to the junction. A custom Perl code was used to extract 4 nucleotides plus the junction from each region from all the detected DelVG in the three replicates of the 6-hpi population. Next the WebLogo platform (49) was used to measure the percentage occurrences of each nucleotide at each position. To decide whether the percentage occurrence differed from what would be expected in the absence of nucleotide enrichment, we generated a random control samples the same way as in the direct repeats. Finally, the two samples: observed/experimental and expected/computational were compared by using two-way analysis of variance (ANOVA). To confirm the validity of this approach, we obtained the same results when we repeated the analysis for the 3-hpi time point and different segments. In addition, we found no significant difference at all nucleotide position upon comparing two random samples, with three replicates each (data not shown).

The percentage length of each segment was calculated based on the total genome length 13,585 nucleotides (e.g., PB2 = 17.2% and NS = 6.5%). Next, the percentage length of each segment was used to calculate the number of junctions per segment based on the total normalized number of junctions of each sample (expected). Finally, these values were compared to the observed values from the actual experiments.

### Data availability.

All NGS data sets generated in this study can be found under BioProject accession number PRJNA725907.
