# Supplementary figures and images for: Influenza A Virus Defective Viral Genomes Are Inefficiently Packaged into Virions Relative to Wild-Type Genomic RNAs
Source: mBio. 2021 Nov 23;12(6):e02959-21. doi: 10.1128/mBio.02959-21 (PMC8609359; doi:10.1128/mBio.02959-21)

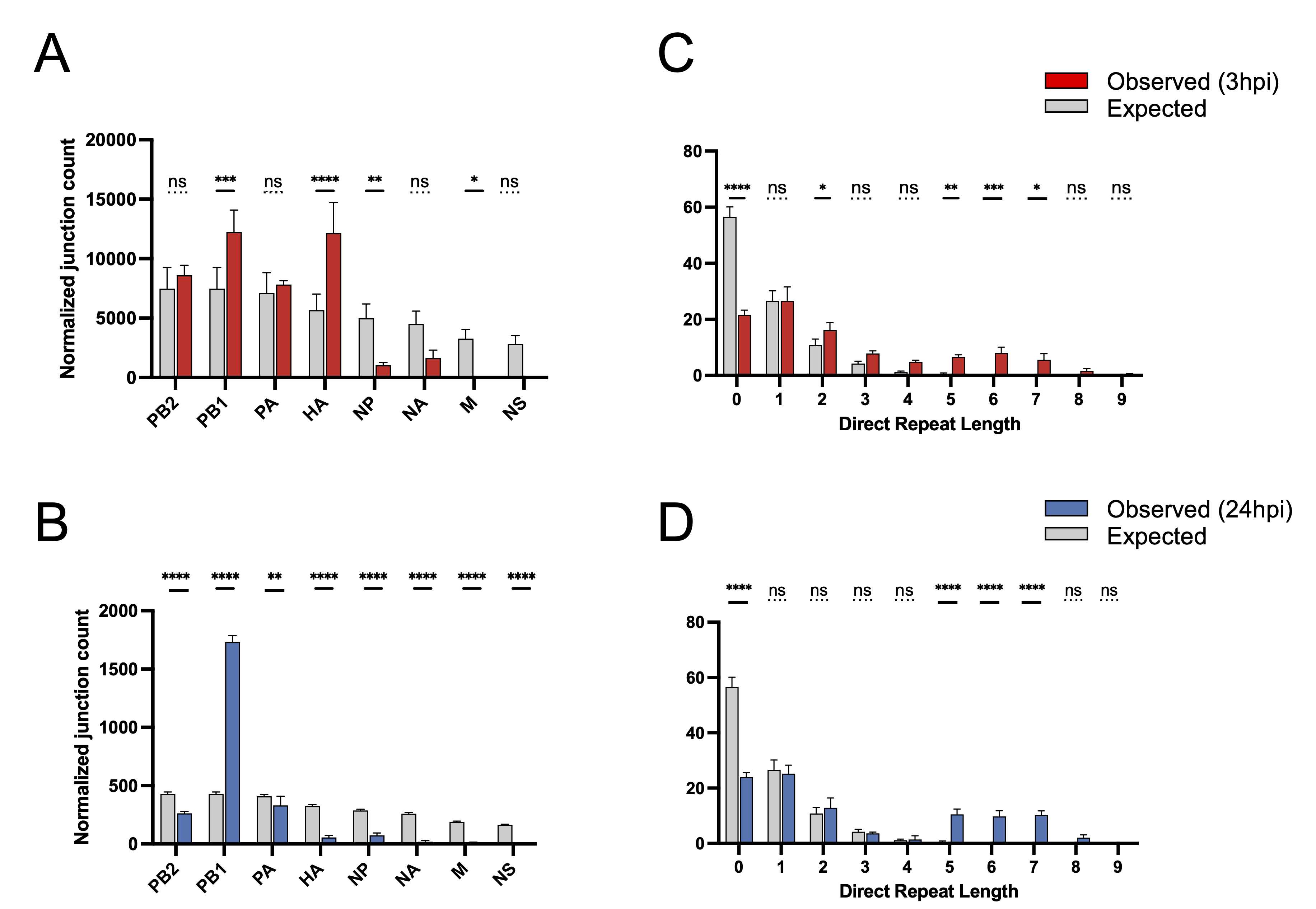

Supplement: FIG S1 [file mbio.02959-21-sf001.tif]

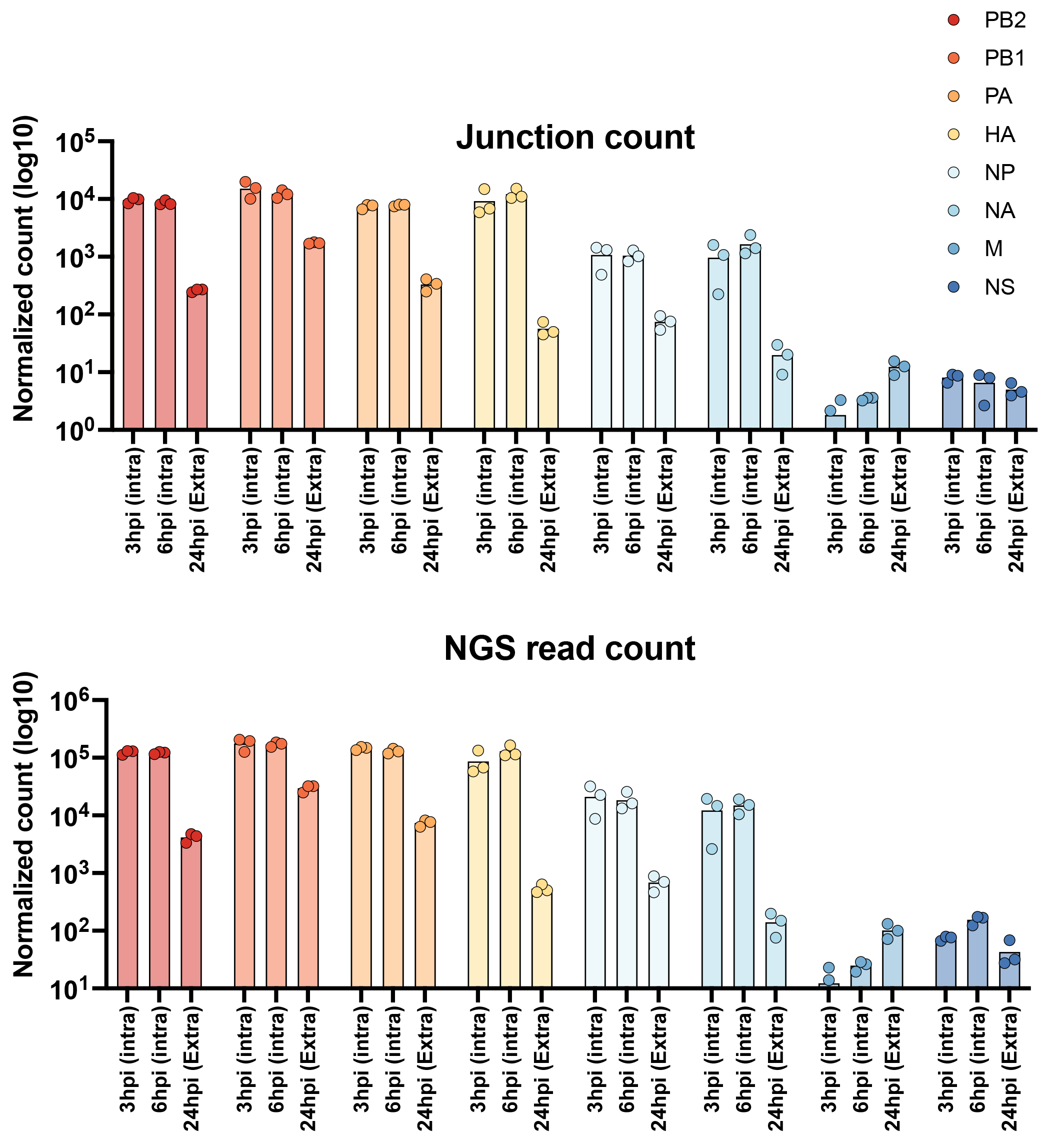

Supplement: FIG S2 [file mbio.02959-21-sf002.tif]

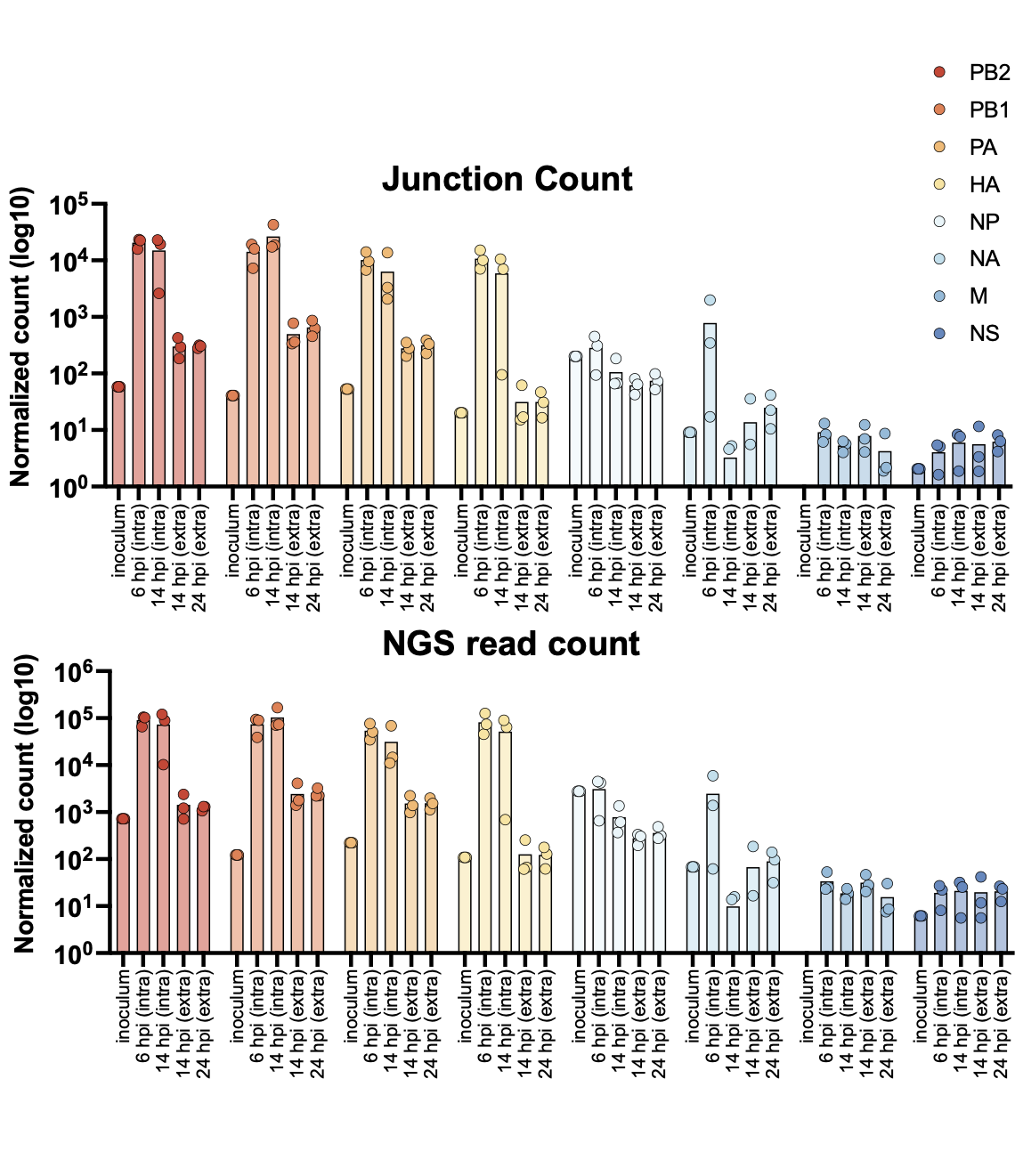

Supplement: FIG S3 [file mbio.02959-21-sf003.tif]

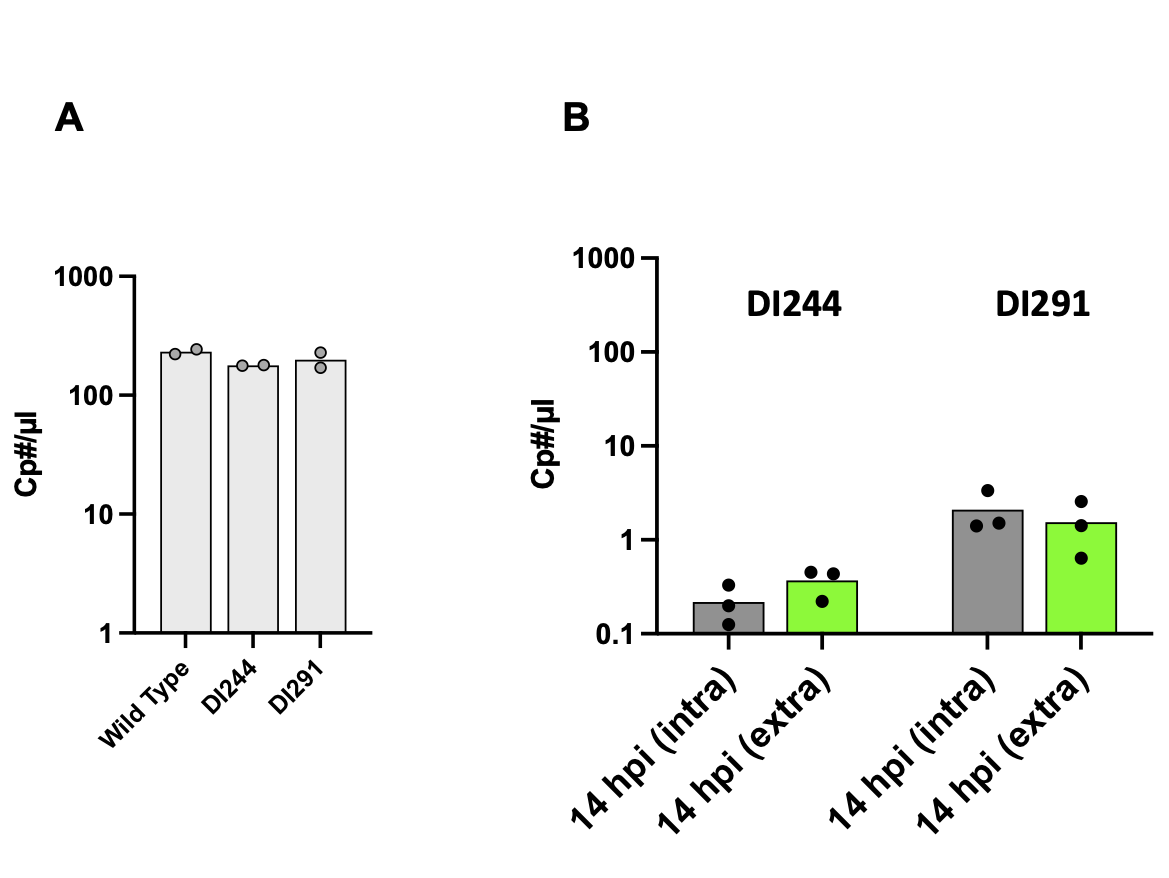

Supplement: FIG S4 [file mbio.02959-21-sf004.tif]

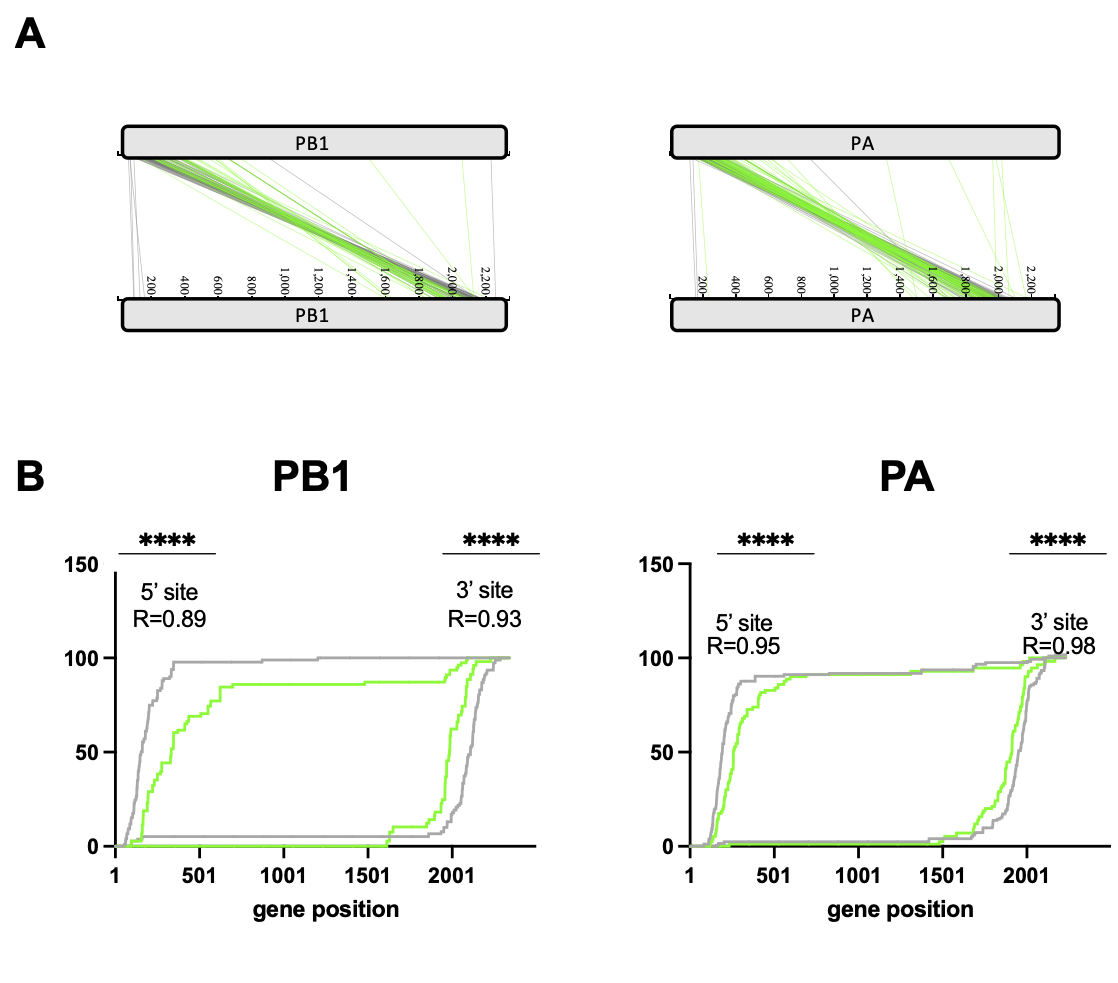

Supplement: FIG S5 [file mbio.02959-21-sf005.tif]
