# Supplementary material for: Influenza A Virus Defective Viral Genomes Are Inefficiently Packaged into Virions Relative to Wild-Type Genomic RNAs
Source: mBio. 2021 Nov 23;12(6):e02959-21. doi: 10.1128/mBio.02959-21 (PMC8609359; doi:10.1128/mBio.02959-21)
Supplement: TABLE S1 [file mbio.02959-21-st001.pdf]

Primer/prob locations	Primer/prob names sequences	Dye
PB2-WT FW-primer (920-940)	TTAGGCAGAACCCAACAGAAG	5'Yakima yellow/ZEN/3'IBFQ
PB2-WT reverse-primer (1040-1020)	ACTGATGATCCGCTTGTTCTC	
PB2-WT Probe (956-979)	TATGCAAGGCTGCAATGGGACTGA	
PB2-DI244 forward-primer (92-109)	AAACCACCGTGGACCATA	5'FAM/ZEN/3'IBFQ
PB2-DI244 Reverse-primer (2169-2148)	GCTCAGTTCATTGATGCTTAGT	
PB2-DI244 Probe (135-151 and 2098-2103)	AGGAAGACAGGAGAAGACTGAGG	
PB2-DI291 forward-primer (281-302)	GATCAGACCGAGTGATGGTATC	
PB2-DI291 Reverse-primer (2169-2147)	GCTCAGTTCATTGATGCTTAGTG	
PB2-DI291 Probe (317-333 and 2051-2062)	CATGGTGGAATAGGAATTAACTGAAGACC	

Table 1: qPCR primers and probes used for the competition assay
